# Editorial Comment: Penile Transplantation: Lessons Learned and Technical Considerations

**DOI:** 10.1590/S1677-5538.IBJU.2022.04.02

**Published:** 2022-07-19

**Authors:** Luciano A. Favorito, Natasha T. Logsdon

**Affiliations:** 1 Unidade de Pesquisa Urogenital Universidade do Estado do Rio de Janeiro Rio de Janeiro RJ Brasil Unidade de Pesquisa Urogenital - Universidade do Estado do Rio de Janeiro - Uerj, Rio de Janeiro, RJ, Brasil

Isabel V Lake ^1^,Alisa O Girard ^1^, Christopher D Lopez ^1^, Damon S Cooney ^1^, Arthur L Burnett ^2, 3^, Gerald Brandacher ^1^, Byoung Chol Oh ^1^, Richard J Redett ^1^

^1^Department of Plastic and Reconstructive Surgery, Johns Hopkins University School of Medicine, Baltimore, Maryland;^2^Department of Urology, Johns Hopkins University School of Medicine, Baltimore, Maryland;^3^ Department of Oncology, Johns Hopkins University School of Medicine, Baltimore, Maryland

J Urol. 2022 May;207(5):960-968.

DOI: 10.1097/JU.0000000000002504 | ACCESS: 35239430

## COMMENT

Penile amputation is an extremely serious and crippling condition with terrible repercussions for patients. Reconstruction techniques are little studied in the literature due to the limited number of cases and the results are not very reproducible ( 1 , 2 ). Penile transplantation is a very good option although there are few reports of success. The present paper is very important and shows a very nice review about penile transplantation. During the paper we can observe the great importance of penile anatomy knowledge for this surgery. The paper has amazing original schematic drawings about penile vascular anatomy.

This paper shows the penile arteries collateral circulation and the vascularization of the perineal skin - key points to the success of penile transplantation. The authors show the vascular anastomosis of the 4 cases of penile transplantation in literature in a very beautiful figure.

The paper concludes that penile allotransplantation represents a revolutionary technique in the management of penile loss. The inclusion of external pudendal artery anastomoses appears to have prevented any form of penile skin necrosis and anastomosis of the corpora cavernosa appears sufficient for restoration of erectile function independent of the cavernous artery.

## References

[1] Wang P, Luo Y, Li YF, Zhang Y, Bi G, Jin DC, et al. Microscopic Replantation of Complete Penile Amputation With Video Demonstration. Urology. 2022:S0090-4295(22)00225-4. 10.1016/j.urology.2022.03.006 35300997

[2] Wu Q, Liu L, Yang Z, Ma N, Wang W, Li YQ. Significance and Surgical Options for Nontranssexual Phalloplasty: A Retrospective Single-Center Analysis of 166 Patients. Ann Plast Surg. 2022;88:440-5. 10.1097/SAP.0000000000003031 34711727

